# Comparative efficacy and safety of analgesics for acute renal colic: A network meta-analysis protocol

**DOI:** 10.1097/MD.0000000000014709

**Published:** 2019-03-08

**Authors:** Shimin Fu, Kebiao Zhang, Manping Gu, Zhiping Liu, Wenzhuo Sun, Mingzhao Xiao

**Affiliations:** a Department of Emergency; b Department of Endocrinology; c Department of Urinary Surgery, The First Affiliated Hospital of Chongqing Medical University, Chongqing, China.

**Keywords:** analgesics, network meta-analysis, protocol, renal colic

## Abstract

**Introduction:**

Acute renal colic is one of the most common urological emergencies. While previous randomized controlled trials (RCTs) and pairwise meta-analyses only looked at the efficacy of 1 or 2 analgesics. It is not fully understood that the comprehensive ranking of the effectiveness and safeness of analgesics from these published articles. Therefore, this network meta-analysis (NMA) aims to compare and rank the different analgesics for treatment of acute renal colic.

**Methods and analysis:**

We will perform a systematic literature search in PubMed, EMBASE, CINAHL, Web of Science, and Cochrane Library to identify RCTs of different analgesics for acute renal colic. RCTs assessing active analgesics intervention against active comparator or placebo controls for acute renal colic will be included. We will also screen the reference lists of included studies, previous reviews and meta-analyses to identify other relevant trials. The primary outcomes will be pain variance at 30 minutes, need rescue medicine, complete pain relief or at least 50% pain relief at 30 minutes, and pain relapse within 24 hours. We will also assess secondary outcomes for safeness (side effects: dizziness, vomit, allergic, hypotension, cardiac toxicity, and drug dependence). The risk of bias of included RCTs will be assessed by using the Cochrane Collaboration's tool, and the quality of evidence will be assessed by using the Grading of Recommendations Assessment, Development and Evaluation (GRADE) instrument. We will perform pairwise meta-analysis and Bayesian NMA to compare the effectiveness and safeness of different analgesic interventions.

**Results:**

This NMA will compare and rank the different analgesics for treatment of acute renal colic.

**Conclusion:**

This is the first systematic review to use the NMA to comprehensively compare and rank analgesics for relieving pain of acute renal colic in adults based on most important factors deciding the choice of initial analgesia, and the results can provide implications for clinical practice and further research.

## Abstract 2

Supplemental Digital Content is available in the text

## 1 Introduction

Renal colic is a severe pain caused by the passage of stones through the urinary system, and it is one of the most common diseases of urological for emergency department visits.^[1,2]^ The reported prevalence of urinary stones varies from 10% to 12% in the industrialized countries.^[3]^ While, in China, it was reported as 1% to 5%, that is more frequent in South than North.^[4]^ One over 10 of the people in the world is living with renal colic,^[5]^ approximately 9% of people experience renal colic annually in America.^[6]^ Classically, acute renal colic presents as the acute severe pain diffusing from the flank to the groin. The pain is often described as the worst pain the patient has ever experienced and may be associated with microscopic hematuria, nausea, and vomit. Renal colic is due to ureteral obstruction by stones, which leads to changes of renal blood flow, intraluminal pressure, and glomerular filtration rate. These changes make up by prostacyclin and prostaglandin E2. Therefore, prostacyclin and prostaglandin E2 play an important role in the creation of renal colic.^[7]^

The excruciating pain demands an effective analgesia, and the target of pharmacotherapy is to provide quick, effective, and safe pain relief in the emergency department. Common analgesics mainly include nonsteroidal anti-inflammatory drugs (NSAIDs), opioids, serotonin 3 antagonists, and paracetamol. NSAIDs and opioids are most commonly used analgesics according to the European Association of Urology (EAU).^[8]^ Some researchers think that NSAIDs and opioids are similar in terms of therapeutic effects and side effects,^[9]^ but some studies found that opioids have more side effects such as respiratory depression and hypotension than other analgesics.^[10,11]^ Some other researchers believe that lidocaine is a new option for renal colic and it may reduce opioid abuse.^[12]^ In addition, combination of drugs is also commonly used to relieve acute renal colic. While there is no comprehensive ranking of the effectiveness and safeness of analgesics or multiple drug combination therapies from published articles. Preference and clinical experience of doctors are main bases of pain management for acute renal colic.^[13]^ Previous randomized controlled trials (RCTs),^[14,15]^ systematic reviews and meta-analyses^[16–19]^ have compared only a few drugs or have addressed exclusively the effectiveness of analgesics while not concern the safeness of analgesics (Table 1). Take a recent systematic review and meta-analysis^[19]^ have been published, it only included NSAIDs, opioids, and paracetamol while not include cholinergic receptor blockers or tramadol. What is more, it performed the NSAIDs versus opioids and NSAIDs versus paracetamol, while not complete the comparison of opioids and paracetamol.^[20]^

**Table 1:**
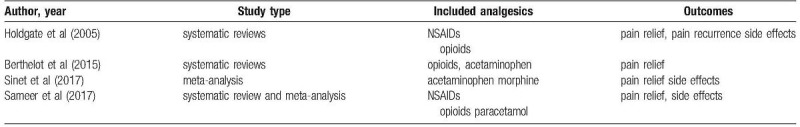
Systematic reviews and meta-analyses of analgesics for acute renal colic.

To fill this gap, we plan to perform a systematic review and network meta-analysis (NMA) of RCTs in adults with renal colic, using data from published studies and unpublished data. NMA is a methodological approach which allows simultaneous comparison of multiple analgesic interventions within a single analysis while preserving randomization. This approach will be used to integrate direct evidence (from studies directly comparing interventions) with indirect evidence (information about 2 treatments derived via a common comparator) from multiple analgesic comparisons to estimate and rank the effectiveness and safeness across all analgesic interventions.^[21]^ Compared with pairwise meta-analysis, it has been found to increase the precision of the estimated effect size.^[22]^

## 2 Methods and analyses

### 2.1 Design

Systematic review and NMA.

### 2.2 Patient and public involvement

Clinical patients or public are not involved in this study because all data is provided by published RCTs.

### 2.3 Eligibility criteria

#### 2.3.1 Types of studies

Only RCTs will be included in this study for limiting potential bias. RCTs assessing active analgesic interventions against active comparator or placebo controls for acute renal colic will be included in this NMA. While quasi-randomized trials will not be included in this study.

#### 2.3.2 Participants

Adults (≥16 years) with a clinical diagnosis of acute renal colic (pain less than 24 hours) in the emergency department will be included in this study. Eligible pregnant women are also included in this study. Patients have a history of analgesic dependence or chronic pain such as cancer pain will be excluded.

#### 2.3.3 Interventions

Analgesics commonly used in renal colic (e.g., diclofenac, indomethacin, ibuprofen, morphine, meperidine, tramadol, atropine, anisodamine, and acetaminophen) and other drugs have the efficacy of analgesia and spasmolysis will be included. RCTs comparing any commonly used analgesic and another analgesic or placebo for treatment of acute renal colic in adults will be included. We will also include trials involving combination therapy (combination of multiple analgesics) while studies involving physiotherapy or psychotherapy will be excluded. Trials comparing the same type of analgesic but at different therapeutic dose (flexible or fixed dose) and different administration method will be considered as the same node in the network analysis.

#### 2.3.4 Outcome measures

The primary outcomes include pain variance at 30 minutes, need rescue medicine, complete pain relief or at least 50% pain relief at 30 minutes and pain relapse within 24 hours. Secondary outcomes include side effects (dizziness, vomit, allergic, hypotension, cardiac toxicity, and drug dependence). The pain variance will be assessed by trial-reported pain score using the visual analog scale (VAS).

### 2.4 Literature search

This study will be conducted following the Preferred Reporting Items for Systematic Reviews and Meta-Analyses Protocol (PRISMA-P) statement and the Cochrane Collaboration.^[23,24]^ We will identify relevant trials by searching in EMBASE, PubMed, Cochrane Library, CINAHL, and Web of Science for RCTs comparing active analgesic interventions for acute renal colic up to September 2018.

Two researchers (SF and KZ) will independently screen these 5 electronic databases by the ways of the text word search, subject headings search and boolean calculation search, and use the following terms and keywords:

“renal colic”, “nephric colic”, “nephrocolic”, “kidney colic”, “ureteric colic”, “renal calculus pain”, “renal stone pain”, “ureteric calculus pain”, “ureteric stone pain”, “NSAIDs”, “non steroidal antiinflammatory drug”, “nonsteroid antiinflammatory agent”, “diclofenac”, “diclofenac sodium”, “indomethacin”, “indomethacin”, “indocid”, “ibuprofen”, “motrin”, “opioid”, “opiate”, “morphine”, “morphia”, “morphina”, “morphinium”, “meperidine”, “pethidine”, “meperidine hydrochloride”, “tramadol”, “cholinergic receptor blocker”, “atropina”, “atropine”, “anisodamine”, “anisodaminum”, “acetaminophen”, “paracetamol”, “panadol”, “randomized controlled trial” and “RCT”. There is no any restriction on language during search. An example of search strategy (PubMed) is outlined in Table 2. Search strategies of other 4 databases are provided in Supplementary material (Table S1–S4).

**Table 2:**
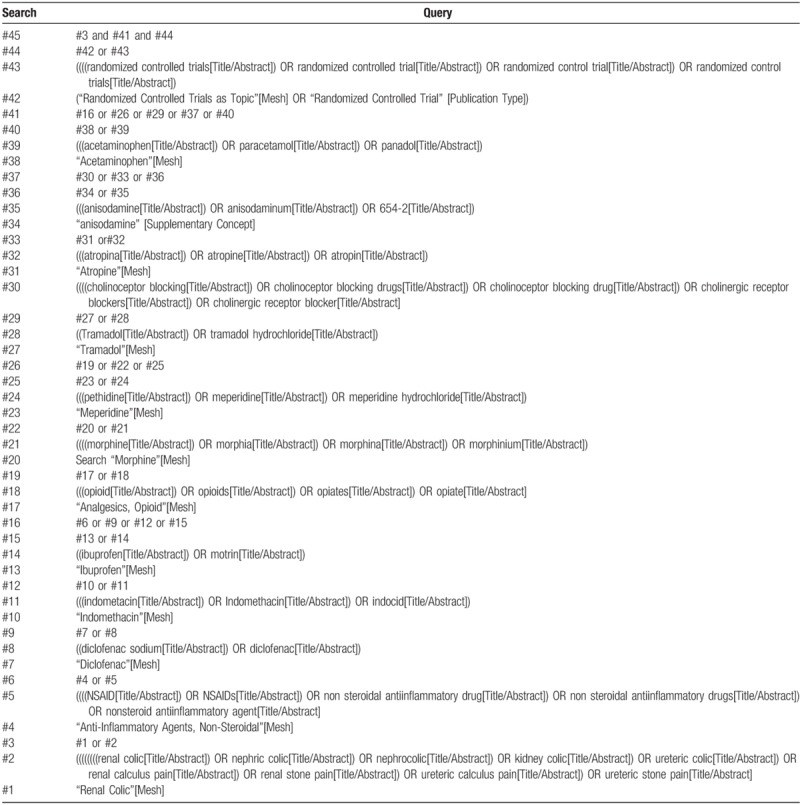
Search strategy of PubMed: up to November 2018 (Continued).

### 2.5 Study selection

We will screen titles and abstracts of records based on inclusion criteria by using Endnote X5 literature management software and review the accuracy and consistency of selections. We will retrieve the full text of studies when there are discrepancies, and discuss whether they should be included. In addition, the reference lists of included studies, previous reviews and meta-analyses will also be screened to identify other relevant articles. Two authors (SF and KZ) will perform this part independently. The process of study selection will be published in a PRISMA flow diagram (Supplementary material).

### 2.6 Data extraction

Two investigators (SF and KZ) will independently extract relevant data from each eligible study and place it into an electronic data-extraction sheet. The major information will be extracted include;

(1) first author, publication year, country,
(2) number of patients, intervention, and control details (drugs, administration methods, dosages),
(3) outcomes (effectiveness and side effects).

We will contact the first author to verify relevant data and request the missing data.

### 2.7 Transitivity and consistency assessment

Transitivity and consistency are important factors influencing the reliability of the conclusion of NMA. The transitivity refers to the similarity of participants, interventions and trial methodology among studies. The sets of studies for each intervention must be similar in their distribution of effect modifiers to permit conclusions based on an NMA combining indirect and direct evidence. We will construct summary tables by pairwise comparisons to assess methodological and clinical similarities of the studies and their populations. In the NMA, the consistency refers to the agreement between direct and 1 or more indirect sources of evidence in a closed loop of studies. We will assess the consistency by comparing model fit from a consistency and independent mean effects model and by informally comparing output from the NMA versus estimates from the pairwise meta-analysis.

### 2.8 Risk of bias assessment

Four investigators (SF, KZ, WS, and ZL) will assess the risk of bias in studies by using the Cochrane risk of bias tool,^[25]^ and the assessment will only be limited to the primary outcomes. Each study will be assessed and scored as “low”, “unclear”, or “high” risk of bias by the following criteria: random sequence generation; allocation concealment; blinding of participants and personnel; blinding of outcome assessment; incomplete outcome data; selective reporting; and other bias.^[26]^ Thus, study with low risk of bias in all key domains will be considered as at low risk of bias, study with high risk of bias in 1 or more key domains will be considered as at high risk of bias, otherwise, it will be considered as at unclear risk of bias.^[27]^

### 2.9 Pairwise meta-analysis

We will perform the pairwise meta-analysis with a random-effects model with STATA (version 12.0). Only studies that provided direct comparison data (e.g., analgesic vs analgesic, analgesic vs placebo, combination of multiple analgesics vs analgesic, combination of multiple analgesics vs another combination of multiple analgesics) will be included in pairwise meta-analysis. Standardized mean difference (SMD) with 95% confidence interval (CI) will be calculated for the continuous outcome (pain variance at 30 minutes), and risk ratio (RR) with 95% CI will be calculated for categorical outcomes (complete pain relief or at least 50% pain relief at 30 minutes, need rescue medicine, pain relapse within 24 hours).^[28]^ If the mean or standard deviation (SD) is not available, we will calculate them from other statistical indices described elsewhere or obtain them by contacting authors.^[29]^ Clinical and methodological heterogeneity across studies will be estimated with inconsistency statistics (*I*^*2*^) (*I*^*2*^ ≤50% indicates that there is no significant heterogeneity, and *I*^*2*^ >50% indicates that there is significant heterogeneity).^[30]^

### 2.10 Network meta-analysis

We will complete the random-effects NMA within a Bayesian framework by busing package “GeMTC”^[31]^ in WinBugs software (version 0.14.3),^[32–33]^ and perform further analyses in STATA (version 12.0) and R (version 3.2.2). We will calculate the results of NMA with effect sizes (SMD or RR) and their CIs,^[34]^ and generate samples by using the Markov Chains Monte Carlo (MCMC) method, and run 2 Markov chains simultaneously with different arbitrarily chosen initial values and use non-informative priors for the parameters. We will measure the goodness-of-fit of the model using the deviance information criterion (DIC).^[35]^ In addition, we will estimate the ranking probabilities for all treatments and perform the process using the surface under the cumulative ranking curve (SUCRA). 100% SUCRA values are expected for the best treatment, and 0% SUCRA values are expected for the worst treatment.^[36]^

If necessary (*I*^*2*^ >50%), we will conduct subgroup analysis of data in primary outcomes to explore the sources of heterogeneity according to the administration methods and analgesics dosages, and we will use the Maantel–Haenszel random-effects model if there is no clinical heterogeneity.^[24]^ In addition, we will perform the sensitivity analysis for primary outcomes by omitting trials where high risk of bias rating have been assessed and trials with a sample size less than 50. Publication bias will be estimated by Begg and Egger funnel plot method (*P* <.05 suggests statistically significant for publication bias).^[37,38]^

### 2.11 Evidence quality assessment

We will assess the quality of evidence for primary and secondary outcomes according to the following the Grading of Recommendations Assessment, Development and Evaluation (GRADE) methodology for risk of bias, inconsistency, indirectness, imprecision, and publication bias. Then the quality of the evidence will be classified as high, moderate, low or very low.^[39–41]^ The summary will be constructed by the GRADE system (GRADE version 3.6).

## 3 Ethics and dissemination

No ethical approval is required in this NMA as no confidential data or patient involvement. Findings of this study will provide evidence of effectiveness and safeness of analgesics for acute renal colic in adults, and they will be submitted to a peer-reviewed journal for publication. The findings will also have implications for clinical practice and further research. This study has been registered on the PROSPERO, and the registration number is CRD42018089335.

## Author contributions

MX and MG conceived this study. SF and KZ contributed to the completion of protocol, search strategy, study selection, data extraction, risk of bias assessment, analysis, evidence quality assessment, and manuscript. WS and ZL assisted in protocol revision, assessment the risk of bias, analysis and evidence quality assessment. All authors have approved this final protocol.

**Conceptualization:** Mingzhao Xiao.

**Data curation:** Shimin Fu, Kebiao Zhang, Wenzhuo Sun.

**Formal analysis:** Shimin Fu, Kebiao Zhang.

**Funding acquisition:** Mingzhao Xiao.

**Methodology:** Zhiping Liu.

**Project administration:** Shimin Fu, Kebiao Zhang.

**Resources:** Mingzhao Xiao.

**Software:** Shimin Fu, Kebiao Zhang.

**Supervision:** Manping Gu.

**Validation:** Zhiping Liu.

**Writing – original draft:** Shimin Fu, Kebiao Zhang.

**Writing – review & editing:** Manping Gu, Mingzhao Xiao.

Shimin Fu orcid: 0000-0002-1977-5469.

## Supplementary Material

Supplemental Digital Content
