# Supplementary material for: Comparative efficacy and safety of analgesics for acute renal colic: A network meta-analysis protocol
Source: Medicine (Baltimore). 2019 Mar 8;98(10):e14709. doi: 10.1097/MD.0000000000014709 (PMC6417635; doi:10.1097/MD.0000000000014709)
Supplement: Supplemental Digital Content [file medi-98-e14709-s001.docx]

| Table S1. Search strategy of Web of Science. | |
| --- | --- |
| Search | Query |
| #9 | #8 AND #7 AND #1 |
| #8 | ((TOPIC: (randomized control trial) OR TOPIC: (randomized control trials) OR TOPIC: (randomized controlled trial) OR TOPIC: (randomized controlled trials) |
| #7 | #6 OR #5 OR #4 OR #3 OR #2 |
| #6 | TOPIC: (Acetaminophen) OR TOPIC: (paracetamol) OR TOPIC: (panadol) |
| #5 | TOPIC: (cholinoceptor blocking) OR TOPIC: (cholinoceptor blocking drugs) OR TOPIC: (cholinoceptor blocking drug) OR TOPIC: (cholinergic receptor blocker) OR TOPIC: (cholinergic receptor blockers) OR TOPIC: (atropina) OR TOPIC: (atropine) OR TOPIC: (atropin) OR TOPIC: (654-2) OR TOPIC: (anisodamine) OR TOPIC: (anisodaminum) |
| #4 | TOPIC: (Tramadol) OR TOPIC: (tramadol hydrochloride) |
| #3 | TOPIC: (Opioid) OR TOPIC: (Opioids) OR TOPIC: (opiates) OR TOPIC: (opiate) OR TOPIC: (morphine) OR TOPIC: (morphia) OR TOPIC: (morphina) OR TOPIC: (morphinium) OR TOPIC: (pethidine) OR TOPIC: (meperidine) OR TOPIC: (meperidine hydrochloride) |
| #2 | TOPIC: (non steroidal antiinflammatory drug) OR TOPIC: (non steroidal antiinflammatory drugs) OR TOPIC: (NSAID) OR TOPIC: (NSAIDs) OR TOPIC: (nonsteroid antiinflammatory agent) OR TOPIC: (diclofenac sodium) OR TOPIC: (diclofenac) OR TOPIC: (indometacin) OR TOPIC: (Indomethacin) OR  TOPIC: (indocid) OR TOPIC: (ibuprofen) OR TOPIC: (motrin) |
| #1 | TOPIC: (renal colic) *OR* TOPIC: (nephric colic) *OR* TOPIC: (nephrocolic) *OR* TOPIC: (kidney colic) *OR* TOPIC: (ureteric colic) *OR* TOPIC: (renal calculus pain) *OR* TOPIC: (renal stone pain) *OR* TOPIC: (ureteric calculus pain) *OR* TOPIC: (ureteric stone pain) |

| Table S2. Search strategy of EMBASE. | |
| --- | --- |
| Nomber | Query |
| #45 | #3 AND #43 AND #44 |
| #44 | #16 OR #26 OR #29 OR #37 OR #40 |
| #43 | #41 OR #42 |
| #42 | 'randomized controlled trial':ab,ti OR 'randomized controlled trials':ab,ti OR 'randomized control trial':ab,ti OR 'randomized control trials':ab,ti |
| #41 | 'randomized controlled trial'/exp |
| #40 | #38 OR #39 |
| #39 | acetaminophen':ab,ti OR 'paracetamol':ab,ti OR 'panadol':ab,ti |
| #38 | 'paracetamol'/exp |
| #37 | #30 OR #33 OR #36 |
| #36 | #34 OR #35 |
| #35 | 'anisodamine':ab,ti OR 'anisodaminum':ab,ti OR '654-2':ab,ti |
| #34 | 'anisodamine'/exp |
| #33 | #31 OR #32 |
| #32 | 'atropina':ab,ti OR 'atropines':ab,ti OR 'atropin':ab,ti |
| #31 | 'atropine'/exp |
| #30 | 'cholinoceptor blocking':ab,ti OR 'cholinoceptor blocking drugs':ab,ti OR 'cholinoceptor blocking drug':ab,ti OR 'cholinergic receptor blockers':ab,ti OR 'cholinergic receptor blocker':ab,ti |
| #29 | #27 OR #28 |
| #28 | 'tramadol':ab,ti OR 'tramadol hydrochloride':ab,ti |
| #27 | 'tramadol'/exp |
| #26 | #19 OR #22 OR #25 |
| #25 | #23 OR #24 |
| #24 | 'meperidine':ab,ti OR 'pethidine':ab,ti OR 'meperidine hydrochloride':ab,ti |
| #23 | 'pethidine'/exp |
| #22 | #20 OR #21 |
| #21 | 'morphine':ab,ti OR 'morphia':ab,ti OR 'morphina':ab,ti OR 'morphinium':ab,ti |
| #20 | 'morphine'/exp |
| #19 | #17 OR #18 |
| #18 | 'opioid':ab,ti OR 'opioids':ab,ti OR 'opiates':ab,ti OR 'opiate':ab,ti |
| #17 | 'opiate'/exp |
| #16 | #6 OR #9 OR #12 OR #15 |
| #15 | #13 OR #14 |
| #14 | 'apo-ibuprofen':ab,ti OR 'motrin':ab,ti |
| #13 | 'ibuprofen'/exp |
| #12 | #10 OR #11 |
| #11 | 'indometacin':ab,ti OR 'indomethacin':ab,ti OR 'indocid':ab,ti |
| #10 | 'indometacin'/exp |
| #9 | #7 OR #8 |
| #8 | 'diclofenac':ab,ti OR 'diclofenac sodium':ab,ti |
| #7 | 'diclofenac'/exp |
| #6 | #4 OR #5 |
| #5 | 'non steroidal antiinflammatory drug':ab,ti OR 'non steroidal antiinflammatory drugs':ab,ti OR 'nsaid':ab,ti OR 'nsaids':ab,ti OR 'nonsteroid antiinflammatory agent':ab,ti |
| #4 | 'nonsteroid antiinflammatory agent'/exp |
| #3 | #1 OR #2 |
| #2 | 'renal colic':ab,ti OR 'nephric colic':ab,ti OR 'nephrocolic':ab,ti OR 'kidney colic':ab,ti OR 'ureteric colic':ab,ti OR 'renal calculus pain':ab,ti OR 'renal stone pain':ab,ti OR 'ureteric calculus pain':ab,ti OR 'ureteric stone pain':ab,ti |
| #1 | 'kidney colic'/exp |

| Table S3. Search strategy of Cochrane Library. | |
| --- | --- |
| Nomber | Query |
| #44 | #3 and #39 and #43 |
| #43 | #40 or #41 or #42 |
| #42 | randomized controlled trial:ti,ab,kw or randomized controlled trials:ti,ab,kw or randomized control trial:ti,ab,kw or randomized control trials:ti,ab,kw |
| #41 | MeSH descriptor: [Randomized Controlled Trials as Topic] explode all trees |
| #40 | MeSH descriptor: [Randomized Controlled Trial] explode all trees |
| #39 | #16 or #26 or #29 or #35 or #38 or #45 or #53 or #38 |
| #38 | #36 or #37 |
| #37 | Acetaminophen:ti,ab,kw or paracetamol:ti,ab,kw or panadol:ti,ab,kw |
| #36 | MeSH descriptor: [Acetaminophen] explode all trees |
| #35 | #30 or #33 or #34 |
| #34 | anisodaminum:ti,ab,kw or anisodamine:ti,ab,kw or 654-2:ti,ab,kw |
| #33 | #31 or #32 |
| #32 | atropine:ti,ab,kw or atropina:ti,ab,kw or atropin:ti,ab,kw |
| #31 | MeSH descriptor: [Atropine] explode all trees |
| #30 | cholinoceptor blocking:ti,ab,kw or cholinoceptor blocking drugs:ti,ab,kw or cholinoceptor blocking drug:ti,ab,kw or cholinergic receptor blockers:ti,ab,kw or cholinergic receptor blocker:ti,ab,kw |
| #29 | #27 or #28 |
| #28 | Tramadol:ti,ab,kw or tramadol hydrochloride:ti,ab,kw |
| #27 | MeSH descriptor: [Tramadol] explode all trees |
| #26 | #19 or #22 or #25 |
| #25 | #23 or #24 |
| #24 | pethidine:ti,ab,kw or meperidine:ti,ab,kw or meperidine hydrochloride:ti,ab,kw |
| #23 | MeSH descriptor: [Meperidine] explode all trees |
| #22 | #20 or #21 |
| #21 | morphine:ti,ab,kw or morphia:ti,ab,kw or morphina:ti,ab,kw or morphinium:ti,ab,kw |
| #20 | MeSH descriptor: [Morphine] explode all trees |
| #19 | #17 or #18 |
| #18 | "opioid":ti,ab,kw or opioids:ti,ab,kw or opiates:ti,ab,kw or opiate:ti,ab,kw |
| #17 | MeSH descriptor: [Opiate Alkaloids] explode all trees |
| #16 | #6 or #9 or #12 or #15 |
| #15 | #13 or #14 |
| #14 | ibuprofen:ti,ab,kw or motrin:ti,ab,kw |
| #13 | MeSH descriptor: [Ibuprofen] explode all trees |
| #12 | #10 or #11 |
| #11 | indometacin:ti,ab,kw or Indomethacin:ti,ab,kw or indocid:ti,ab,kw |
| #10 | MeSH descriptor: [Indomethacin] explode all trees |
| #9 | #7 or #8 |
| #8 | diclofenac:ti,ab,kw or diclofenac sodium:ti,ab,kw |
| #7 | MeSH descriptor: [Diclofenac] explode all trees |
| #6 | #4 or #5 |
| #5 | NSAID:ti,ab,kw or NSAIDs:ti,ab,kw or non steroidal antiinflammatory drug:ti,ab,kw or non steroidal antiinflammatory drugs:ti,ab,kw or nonsteroid antiinflammatory agent:ti,ab,kw |
| #4 | MeSH descriptor: [Anti-Inflammatory Agents, Non-Steroidal] explode all trees |
| #3 | #1 or #2 |
| #2 | Renal Colic:ti,ab,kw or nephric colic:ti,ab,kw or nephrocolic:ti,ab,kw or kidney colic:ti,ab,kw or ureteric colic:ti,ab,kw or renal calculus pain:ti,ab,kw or renal stone pain:ti,ab,kw or ureteric calculus pain:ti,ab,kw or ureteric stone pain:ti,ab,kw |
| #1 | MeSH descriptor: [Renal Colic] explode all trees |

| Table S4. Search strategy of CINAHL. | |
| --- | --- |
| Search | Query |
| S41 | S1 AND S37 AND S40 |
| S40 | S38 OR S39 |
| S39 | randomized control trial OR randomized control trials OR randomized controlled trial OR randomized controlled trials |
| S38 | (MH "Randomized Controlled Trials") |
| S37 | S14 OR S27 OR S33 OR S36 |
| S36 | S34 OR S35 |
| S35 | Acetaminophen) OR paracetamol OR panadol |
| S34 | (MH "Acetaminophen") |
| S33 | S28 OR S31 OR S32 |
| S32 | 654-2 OR anisodamine OR anisodaminum |
| S31 | S29 OR S30 |
| S30 | atropina OR atropine OR atropin |
| S29 | (MH "Atropine") |
| S28 | cholinoceptor blocking OR cholinoceptor blocking drugs OR cholinoceptor blocking drug OR cholinergic receptor blocker OR cholinergic receptor blockers |
| S27 | S25 OR S26 |
| S26 | Tramadol) OR tramadol hydrochloride |
| S25 | (MH "Tramadol") |
| S24 | S17 OR S20 OR 23 |
| S23 | S21 OR S22 |
| S22 | pethidine OR meperidine OR meperidine hydrochloride |
| S21 | (MH "Meperidine") |
| S20 | S18 OR S19 |
| S19 | morphine OR morphia OR morphina OR morphinium |
| S18 | (MH "Morphine**+**") |
| S17 | S15 OR S16 |
| S16 | Opioid OR Opioids OR opiates OR opiate |
| S15 | (MH "Analgescis, Opioid**+**") |
| S14 | S4 OR S7 OR S10 OR S13 |
| S13 | S11 OR S12 |
| S12 | ibuprofen OR motrin |
| S11 | (MH "ibuprofen") |
| S10 | S8 OR S9 |
| S9 | indometacin OR  Indomethacin OR indocid |
| S8 | (MH "indometacin") |
| S7 | S5 OR S6 |
| S6 | diclofenac sodium OR  diclofenac |
| S5 | (MH "diclofenac") |
| S4 | S2 OR S3 |
| S3 | NSAIDs OR NSAID OR non steroidal antiinflammatory drug OR non steroidal antiinflammatory drugs OR nonsteroid antiinflammatory agent |
| S2 | (MH "Antiinflammatory Agents, Nonsteroidal**+**") |
| S1 | renal colic OR nephric colic OR nephrocolic OR kidney colic OR ureteric colic OR renal calculus pain OR renal stone pain OR ureteric calculus pain OR ureteric stone pain |


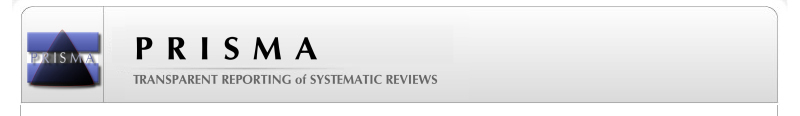
**PRISMA 2009 Flow Diagram**

Studies included in quantitative synthesis (meta-analysis)
(n = )

Studies included in qualitative synthesis
(n = )

Full-text articles excluded, with reasons
(n = )

Full-text articles assessed for eligibility
(n = )

Records excluded
(n = )

Records screened
(n = )

Records after duplicates removed
(n = )

Additional records identified through other sources
(n = )

## Identification

## Eligibility

## Included

## Screening

Records identified through database searching
(n = )
